# Cytokine expression profiles in white blood cells of patients with small fiber neuropathy

**DOI:** 10.1186/s12868-022-00770-4

**Published:** 2023-01-05

**Authors:** Luisa Kreß, Nadine Egenolf, Claudia Sommer, Nurcan Üçeyler

**Affiliations:** Department of Neurology, University of Würzburg, Josef-Schneider-Str. 11, 97080 Würzburg, Germany

**Keywords:** Small fiber neuropathy, Cytokines, White blood cells, Gene expression

## Abstract

**Background:**

The role of cytokines in the pathophysiology, diagnosis, and prognosis of small fiber neuropathy (SFN) is incompletely understood. We studied expression profiles of selected pro- and anti-inflammatory cytokines in RNA from white blood cells (WBC) of patients with a medical history and a clinical phenotype suggestive for SFN and compared data with healthy controls.

**Methods:**

We prospectively recruited 52 patients and 21 age- and sex-matched healthy controls. Study participants were characterized in detail and underwent complete neurological examination. Venous blood was drawn for routine and extended laboratory tests, and for WBC isolation. Systemic RNA expression profiles of the pro-inflammatory cytokines interleukin (IL)-1ß, IL-2, IL-8, tumor necrosis factor-alpha (TNF) and the anti-inflammatory cytokines IL-4, IL-10, transforming growth factor beta-1 (TGF) were analyzed. Protein levels of IL-2, IL-8, and TNF were measured in serum of patients and controls. Receiver operating characteristic (ROC)-curve analysis was used to determine the accuracy of IL-2, IL-8, and TNF in differentiating patients and controls. To compare the potential discriminatory efficacy of single versus combined cytokines, equality of different AUCs was tested.

**Results:**

WBC gene expression of IL-2, IL-8, and TNF was higher in patients compared to healthy controls (IL-2: p = 0.02; IL-8: p = 0.009; TNF: p = 0.03) and discriminated between the groups (area under the curve (AUC) ≥ 0.68 for each cytokine) with highest diagnostic accuracy reached by combining the three cytokines (AUC = 0.81, sensitivity = 70%, specificity = 86%). Subgroup analysis revealed the following differences: IL-8 and TNF gene expression levels were higher in female patients compared to female controls (IL-8: p = 0.01; TNF: p = 0.03). The combination of TNF with IL-2 and TNF with IL-2 and IL-8 discriminated best between the study groups. IL-2 was higher expressed in patients with moderate pain compared to those with severe pain (p = 0.02). Patients with acral pain showed higher IL-10 gene expression compared to patients with generalized pain (p = 0.004). We further found a negative correlation between the relative gene expression of IL-2 and current pain intensity (p = 0.02). Serum protein levels of IL-2, IL-8, and TNF did not differ between patients and controls.

**Conclusions:**

We identified higher systemic gene expression of IL-2, IL-8, and TNF in SFN patients than in controls, which may be of potential relevance for diagnostics and patient stratification.

## Background

Small fiber neuropathy (SFN) affects the small caliber A-delta and C-fibers [1]. SFN patients typically report acral or generalized burning pain and par- and dysesthesias [1]. While the underlying pathomechanism remains unclear, inflammatory processes and processes of nociceptor degeneration and sensitization may be of relevance [2]. There is growing evidence for the induction of nociceptor degeneration by local inflammation, oxidative stress following impaired calcium homeostasis, and alteration of the energy metabolism via activation of the axonal caspases [2]. Nociceptor sensitization may result directly from inflammatory processes, which may arise by cytokine or chemokine activation [3]. Basic mechanisms of the inflammatory response and potential underlying pathways are well characterized in neurodegenerative and metabolic disorders [4, 5], while hardly known in peripheral painful and painless neuropathies.

There is evidence for pro- and anti-inflammatory cytokines to act as key contributors to SFN pain by directly targeting cutaneous nociceptors [6]. Locally, we found elevated gene expression levels of the pro-inflammatory cytokines interleukin (IL)-6 and IL-8 in whole skin biopsy samples [7]. Analysis of distinct skin cells showed higher expression levels of IL-6 and IL-8 in fibroblasts and of transforming growth factor beta-1 (TGF) in keratinocytes of SFN patients compared to healthy controls [6]. In addition to a pathophysiological role of cytokines in neuropathic pain and SFN, there is growing evidence for their usage as diagnostic and prognostic parameters [8–10]. While reports exist for inflammatory conditions [11, 12], data in neuropathies are rare [13].

In our previous study, we analyzed the pro-inflammatory cytokines IL-1ß, IL-2, IL-8, tumor necrosis factor-alpha (TNF) and the anti-inflammatory cytokines IL-4, IL-10, and TGF, which are examples of frequently investigated cytokines in painful neuropathies [14]. We found systemically higher expression levels of IL-2, IL-10, and TGF in whole blood samples of SFN patients compared to healthy controls. Here, we followed-up these cytokines in a larger study cohort, provide cellular allocation by investigation on white blood cells (WBC) and investigate data on protein level.

## Methods

### Patients and healthy controls

From 2015 to 2019, 52 patients with a medical history and clinical phenotype indicative of SFN (32 women, 20 men; median age: 54 years; range: 19–73) and 21 age and sex-matched healthy controls (13 women, 8 men; median age: 48 years; range: 22–66) were prospectively recruited as part of a larger study [15]. Detailed medical and pain history, including pain character, intensity (determined on a numeric rating scale [NRS]: range 0 = no pain; 10 = maximum pain imaginable), and localization were recorded. All study participants underwent complete neurological examination and nerve conduction studies to exclude polyneuropathy. Five small fiber-specific tests, namely quantitative sensory testing (QST), recording of pain-related evoked potentials (PREP), corneal-confocal microscopy (CCM), quantitative sudomotor axon reflex test (QSART), and skin punch biopsy were performed as part of a larger study; hence, detailed data on methods, results, and discussion have been published elsewhere [15]. In brief: QST (Somedic, Hörby, Sweden) was performed at the dorsum of the right foot following the standardized protocol of the German Research Network on Neuropathic Pain. PREP were recorded after electrical A-delta fiber stimulation via concentric electrodes (Inomed Medizintechnik GmbH, Lübeck, Germany). CCM was performed using a retinal tomograph (Heidelberg Retina Tomograph Rostock Cornea Module, Heidelberg, Germany). QSART was done on the lateral dorsum of the foot using Q-Sweat (WR Medical Electronics, Maplewood, MA, USA) and following the manufacturer’s protocol. Six-millimeter skin biopsies were taken from the right lateral lower leg and upper thigh to determine the intraepidermal nerve fiber density following a standard protocol [15]. Study participants reporting or diagnosed with polyneuropathy, diabetes mellitus, renal insufficiency, uncontrolled thyroid dysfunction, acute or chronic infection, malignancy within the last five years, substance dependence, regular alcohol consumption, or severe psychiatric disorder were excluded. Due to the prerequisites of individual small fiber tests, the following exclusion criteria were additionally applied: epilepsy, cardiac pacemaker, eye disease or surgery, or usage of hard contact lenses. Healthy controls were further free of any neurological disorder.

### Laboratory blood tests

All patients and controls underwent venous blood drawing under standardized conditions. Blood samples were collected between 8:00 and 9:00 AM after overnight fasting, avoiding heavy physical activity or meals, and alcohol consumption on the previous day. To determine the etiology of SFN and not miss potential exclusion criteria, venous blood was drawn in serum (14 ml) and in ethylenediaminetetraacetic acid (EDTA) monovettes (27 ml) to perform routine and extended laboratory tests, and next-generation gene-panel sequencing. Detailed data on these laboratory tests were published previously [15]. Venous blood was used to perform an oral glucose tolerance test and for WBC and serum isolation.

### Gene expression analysis in white blood cells

#### White blood cell isolation

Eighteen ml venous blood was drawn in EDTA monovettes and incubated (30 min, room temperature (RT)). During incubation, nine 15-ml falcon tubes (Greiner bio-one, Kremsmünster, Austria) were loaded with 7.5 ml erythrocyte lysis (EL)-buffer on ice (Quiagen, Hilden, Germany) and assembled with 3 ml of EDTA blood each. Throughout an incubation period of 25 min, falcon tubes were vortexed twice after 7 min and 15 min. After centrifugation (400 g, 10 min, 4 °C), discarding the supernatant fluid, and resuspending cell pellets in 3 ml EL-buffer, samples were centrifuged again (400 g, 10 min, 4 °C). Subsequently, supernatant was removed, cell pellets were re-suspended in 1.5 ml ribonucleic acid (RNA) protect cell reagent (Quiagen, Hilden, Germany), aliquoted 500 µl each, and frozen at -80 °C before RNA extraction.

#### RNA extraction

For RNA isolation, the miRNeasy Mini Kit (Quiagen, Hilden, Germany) was applied and frozen samples containing WBC RNA and RNA protect cell reagent were used. Samples were suspended in 700 µl of Qiazol Lysis reagent (guanidine thiocyanate and phenol mixture) and incubated for 5 min at RT. Then, 140 µl chloroform (Carl Roth, Karlsruhe, Germany) was added and the samples were shaken vigorously for 15 s. Samples were then incubated for up to 3 min at RT and centrifuged (12.000*g*, 15 min, 4 °C). Afterwards, the upper aqueous phase was discarded and 525 µl 100% ethanol was added. 700 µl of the suspension was then transferred into a silica-membrane RNeasy spin column (supplied with the kit) and centrifuged twice (8.000*g*, 20 s, RT). Three further centrifugation steps (each 8.000*g*, 20 s, RT) using silica-membrane RNeasy spin columns were performed. mRNA was eluted in 30 µl RNAse-free-water. RNA concentration was measured via Nanodrop® spectrophotometer (Peqlab, Erlangen, Germany). To assess RNA purity, the ratio of the absorbance (A) at different wavelengths was calculated. A median ratio of 2.0 for A _260 nm/280 nm_ (range 1.85–2.05) was defined as pure RNA. Samples were stored at − 80 °C before further processing.

#### Reverse transcription PCR

For reverse transcription polymerase chain reaction (PCR), 250 ng RNA was used and sterile distilled water (Braun, Melsungen, Germany) was added to reach a total volume of 32.8 µl each. Samples were supplemented with 5 µl Random Hexamer (TaqMan Reverse Transcription Reagents, Thermo Fisher Scientific, Waltham, MA, USA) and incubated (3 min, 85 °C). Two µl Oligo-DT and 60.2 µl Master Mix (produced of 10 µl 10 × PCR buffer, 6.25 µl multiscribe reverse transcriptase, 2 µl RNase inhibitor, 22 µl MgCl_2_, and 20 µl dNTPs) were added. PCR was run in a PCR-Cycler Advanced Primus 96-PCR (Peqlab Biotechnology, Erlangen, Germany) under the following conditions: annealing (10 min, 25 °C), reverse transcription (60 min, 48 °C), and enzyme inactivation (5 min, 95 °C). cDNA was stored at − 20 °C before further processing. RT-PCR was done shortly before running qRT-PCR. Using cDNA samples ≤ 2 months was ensured. To guarantee high quality, cDNA concentration was measured directly after RT-PCR and before qRT-PCR.

#### Quantitative real-time PCR

We investigated the following gene targets: pro-inflammatory cytokines IL-1ß (Hs00174097_m1), IL-2 (Hs00174114_m1), IL-8 (Hs00174103_m1), and TNF (Hs00174128_m1) and anti-inflammatory cytokines IL-4 (Hs00174122_m1), IL-10 (Hs00174086_m1), and TGF (Hs99999918_m1). Primers were commercially designed and validated primers (TaqMan, Thermo Fisher Scientific, Waltham, MA, USA). A Micro Amp Optical 96-Well Reaction Plate (Thermo Fisher Scientific, Waltham, MA, USA), containing a negative control without cDNA, and a calibrator sample was used to measure the samples. The calibrator sample (determined as the sample with threshold cycle (Ct-) values next to the respective control groups' mean Ct values), was individually assigned to each target gene. The reaction contained: 5 µl cDNA, 2 µl TaqMan Universal Master Mix (Thermo Fisher Scientific, Waltham, MA, USA), 1.75 µl sterile distilled water (Braun, Melsungen, Germany), and 0.25 µl of the target primer. Endogenous control eukaryotic, 18 s RNA (Hs99999901_s1) was used as housekeeping gene as it was validated on human biomaterial in our previous studies [7, 14, 16, 17]. 18 s reaction mixture contained 2.5 µl cDNA, 2 µl TaqMan Universal Master Mix (Thermo Fisher Scientific, Waltham, MA, USA), 4.25 µl sterile, distilled water (Braun, Melsungen, Germany), and 0.25 µl 18sRNA. Target genes were measured as triplicates, 18 s RNA as duplicates. the following three steps: first incubation (50 °C, 2 min), second incubation (95 °C, 10 min), and 40 cycles (95 °C, 15 s and 60 °C, 1 min). The analysis was done by using StepOnePlus™ Cycler (Thermo Fisher Scientific, Waltham, MA, USA). 2^−deltadeltaCt^ method was performed for data evaluation.

### Protein expression analysis of cytokines

#### Serum collection

Nine ml venous blood was drawn in serum monovettes and incubated (30 min, RT). Monovettes were centrifuged (400*g*, 10 min, 4 °C), serum was aliquoted 500 µl each, and frozen at − 80 °C before further use.

#### Enzyme-linked immunosorbent assay

To determine the protein levels of IL-2, IL-8, and TNF in serum of patients and healthy controls, the following enzyme-linked immunosorbent assay (ELISA) kits were used: Invitrogen human IL-2, human IL-8 and human TNF-alpha ELISA kit (each Thermo Fisher Scientific, Waltham, MA, USA). ELISA was performed according to the manufacture’s protocol. The provided analytic sensitivities of the assays were given as follows: 9.1 pg/ml for IL-2, < 5.0 pg/ml for IL-8, and 1.7 pg/ml for TNF.

### Statistical analysis

We used SPSS 26 (IBM Deutschland GmbH, Ethningen, Germany) for statistical analysis. Data were not normally distributed, thus the non-parametric Mann–Whitney-U-test and the Spearmann's test for correlation analysis were applied. Receiver operating characteristic (ROC)-curve analysis was used to calculate the area under the curve (AUC), specificity, sensitivity, and the optimal cut-off value of IL-2, IL-8, TNF and their combination to evaluate the accuracy in differentiating SFN patients and healthy controls. ROC curve plotting and analysis was achieved by using the web-based tool easy ROC (version 1.3.1), which is based on R Langue Environment [18]. Optimal cut-off values were defined by Youden method. Discriminatory efficacy of single cytokines was determined by AUC comparison. DeLong’s test procedure was used to non-parametrically test the hypothesis of the equality of the AUCs of combined cytokines. To perform DeLong’s test R package pROC (version 4.2.1) was used. Scatter and box plots were created with GraphPad Prism 9.1.0.221 software. G*Power version 3.1.9.7 (http://www.psycho.uni-duesseldorf.de/abteilungen/aap/gpower3/) was used for post-hoc sample size calculation. P < 0.05 was considered significant. Post-hoc sample size calculation revealed that n = 84 patients and n = 34 controls should be included in our study assuming a large effect size. With n = 52 SFN patients and n = 21 controls, we performed an exploratory study.

## Results

### Clinical and laboratory data

Baseline clinical and laboratory data of the study cohort is summarized in Table 1. In 22/52 (42%) SFN patients, a potential underlying reason was found and 30/52 (58%) SFN patients were classified as having an idiopathic SFN. Laboratory tests were normal in 43/52 (83%) of the patients except for the following abnormalities: cell count (n = 3), serum (n = 5), elevated HbA1c (n = 7), vitamin B12 deficiency (n = 2), thyroid dysfunction (n = 6), and detection of autoantibodies (n = 2). An impaired glucose tolerance was detected in 15/52 (29%) of the patients. 15/52 (29%) patients reported acral, 22/52 (42%) generalized, and 15/52 (29%) simultaneously acral and generalized pain. The median current pain intensity was 4/10 NRS with a range from 0–8. Female patients reported a mean pain intensity of 5/10 NRS (range 0–8), male patients of 4/10 NRS (range 0–8). In 30/52 (58%) of SFN patients, signs of small fiber impairment were found on neurological examination, namely: thermal hypoesthesia (n = 13), hypo-/hyperalgesia (n = 15), allodynia (n = 4), dys-/paresthesia (n = 7). 29/52 (56%) patients reported autonomic symptoms; dyshidrosis was the most common (n = 29).

**Table 1:** Basic clinical and laboratory data of study population

	Patients (n = 52)	Controls (n = 21)
Age [years] (range)	54^a^ (19–73)	48^a^ (22–66)
Gender (F/M)	32/20	13/8
Time since diagnosis [years] (range)	0.25^a^ (< 1 month-12)	NA
Pain duration [years]	3.25^a^ (< 1 month-24)	NA
Assumed etiology of SFN		NA
Determined (In some patients ≥ 1 pathological finding was present, thus, the sum exceeds 100%)	22/52 (42%)	
Diabetes or impaired glucose tolerance	22/52 (42%)	
Vitamine B12 deficiency	2/52 (4%)	
Hereditary	5/52 (10%)	
Thyroid dysfunction	6/52 (12%)	
Idiopathic	30/52 (58%)	
Abnormal results in routine laboratory tests b (In some patients ≥ 1 pathological finding was present)	9/52 (17%)	Not investigated
Leucocytosis (Ref.: 5–10*10^3^/µl)	2/52 (4%)	
Leucopenia (Ref.: 5–10*10^3^/µl)	1/52 (2%)	
Creatinine (Ref.: 0–0.95 mg/dl) ↑	2/52 (4%)	
CRP (Ref.: 0–0.5 mg/dl) ↑	2/52 (4%)	
Gamma GT (Ref.: < 40 U/l) ↑	1/52 (2%)	
Abnormal results in extended laboratory tests c (In some patients ≥ 1 pathological finding was present)	28/52 (51%)	NA
HbA1c (Ref.: ≤ 6.1%) ↑	7/52 (13%)	
Vitamin B12 (Ref.: ≥ 197 pg/ml) ↓	2/52 (4%)	
TSH (Ref.: 0.3–4.0 mlU/l) ↑	1/52 (2%)	
TSH (Ref.: 0.3–4.0 mlU/l) ↓	1/52 (2%)	
Detected autoantibodies (antinuclear antibodies, extractable nuclear antigen antibodies, anti-neutrophil cytoplasmic antibodies)	2/52 (4%)	
Pathological oGTT (2 h glucose level ≤ 140 mg/dl)	15/52 (29%)	NA
Pain distribution		NA
Acral	15/52 (29%)	
Generalized	22/52 (42%)	
Both	15/52 (29%)	
Pain intensity [NRS] (range)		NA
Current pain intensity	4^a^ (0–8)	
Maximum pain intensity	8^a^ (3–10)	
Mean pain intensity	5^a^ (0–8)	
Female	5^a^ (0–8)	
Male	4^a^ (0–8)	
Signs of small fiber impairment in neurological examination	30/52 (58%)	None
Thermal hypoesthesia	13/52 (25%)	
Hypo-/hyperalgesia	15/52 (29%)	
Allodynia	4/52 (8%)	
Dysesthesia/paresthesia	7/52 (13%)	
Additional symptoms		NA
Gastrointestinal symptoms	3/52 (5%)	
Obstipation	1/52 (2%)	
Diarrhea	2/52 (4%)	
Autonomic symptoms	29/52 (56%)	
Hypo-/hyperhidrosis	27/52 (52%)	
Sexual dysfunction	7/52 (13%)	
Impairment of micturition	6/52 (12%)	
Repetitive syncope	0/52 (0%)	

*CRP* C-reactive protein, *F* female, *HbA1c* hemoglobin A1c, *IENFD* intraepidermal nerve fiber density, *M* male, *NA* not applicable, *NRS* numeric rating scale, *oGTT* oral glucose tolerance test, *Ref.* reference, *SFN* small fiber neuropathy, *TSH* thyroid stimulating hormone, *WBC* white blood cells

^a^Data are given as median

^b^Individual data: leucocytosis: 12.0*10^3^/µl; 15.6*10^3^/µl; leucopenia: 3.6*10^3^/µl; creatinine ↑: 0.97 mg/dl; 1.0 mg/dl; CRP ↑: 0.84 mg/dl; 2.47 mg/dl; gamma GT ↑: 86.2 U/l

^c^Individual data: HbA1c ↑: 6.2% (3x); 6.3%; 6.6%; 6.9%; 7.7%; vitamin B12 ↓: 137 pg/ml; 195 pg/ml; TSH ↑: 9.2 mlU/l; TSH ↓: 0.1 mlU/l; detected autoantibodies: antinuclear antibodies 1:80; antinuclear antibodies 1:160

### Gene expression data

#### Higher expression of IL-2, IL-8, and TNF in WBC of SFN patients compared to controls

**Fig. 1:**
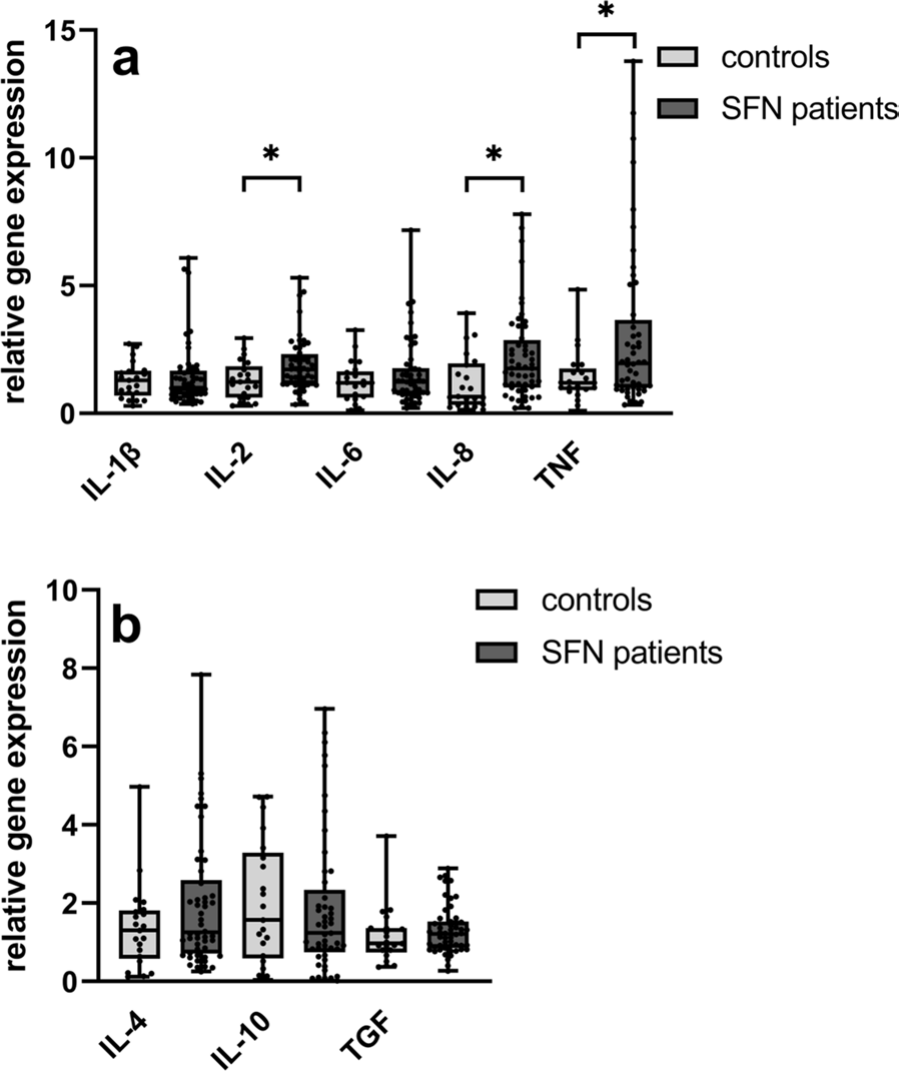
HYPERLINK "sps:id::fig1||locator::gr1||MediaObject::0" Gene expression of cytokines in WBC of SFN patients compared to controls. The scatter plots show the gene expression of pro- (IL-1ß, IL-2, IL-6, IL-8, and TNF) (a) and anti- (IL-4, IL-10, and TGF) inflammatory cytokines. Gene expression of pro-inflammatory cytokines IL-2, IL-8, and TNF was higher in WBC in patients compared to controls. Anti-inflammatory cytokine expression did not differ between groups (b). Number of samples investigated: SFN patients = 52; controls = 21. *IL*− interleukin, *SFN* small fiber neuropathy, *TGF* transforming growth factor beta-1, *TNF* tumor necrosis factor-alpha. *p < 0.05

Gene expression of the pro-inflammatory cytokines IL-2, IL-8, and TNF was higher in patients (n = 52) compared to controls (n = 21) (IL-2: p = 0.02; IL-8: p = 0.009; TNF: p = 0.03) (Fig. 1a). We did not find intergroup differences in the gene expression levels of the pro-inflammatory cytokines IL-1ß and IL-6 (Fig. 1a) and the anti-inflammatory cytokines IL-4, IL-10, and TGF (Fig. 1b).

#### Highest accuracy to distinguish SFN patients and healthy controls combining IL-2, IL-8, and TNF

**Fig. 2:**
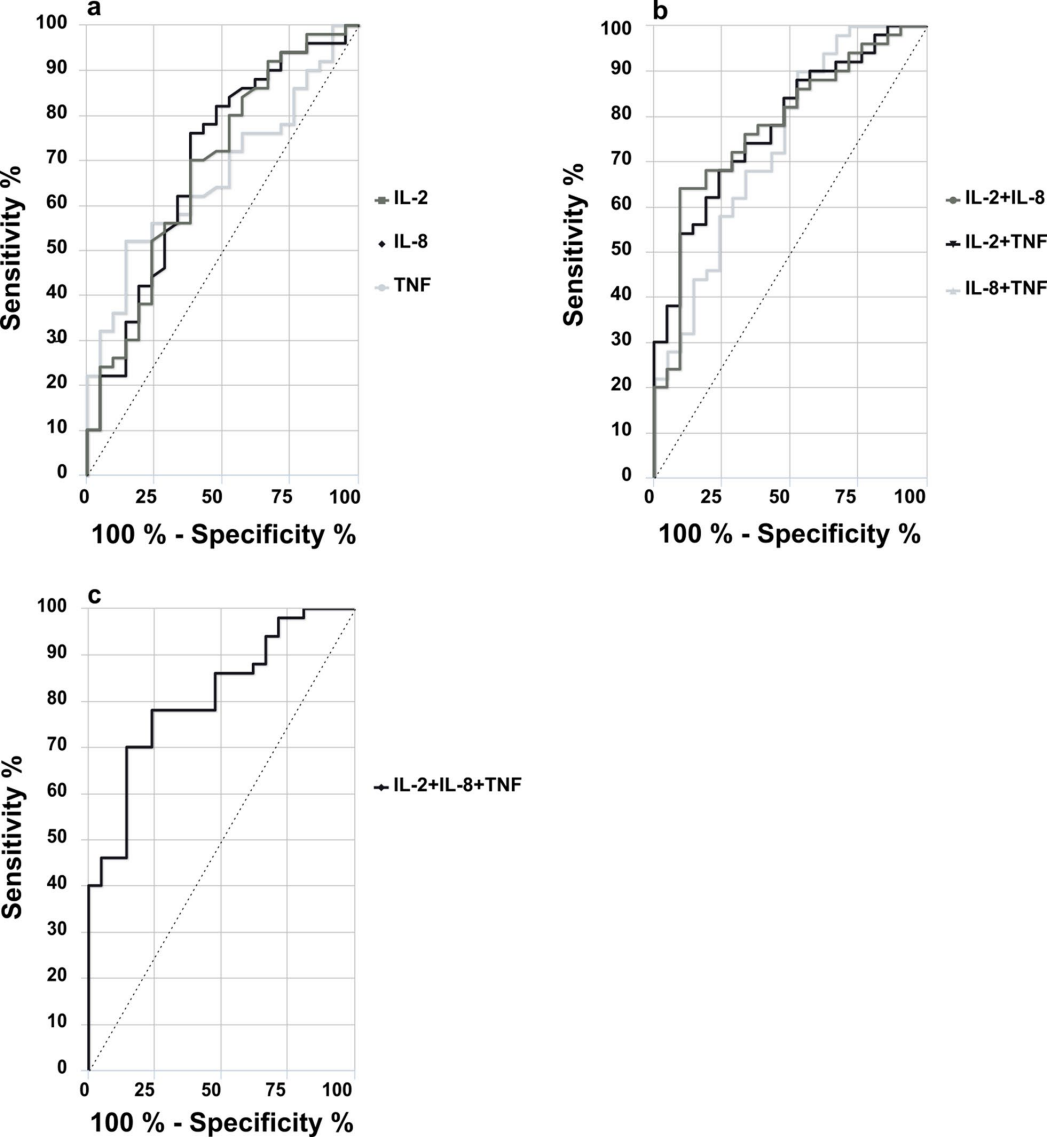
ROC-curve analysis of cytokine gene expression levels in WBC of SFN patients compared to controls. The ROC-curves show the total AUC for IL-2, IL-8, and TNF separately (**a**), after combination of two out of three cytokines (**b**), and after merging IL-2, IL-8, and TNF together (**c**). AUC was highest for IL-8 (AUC = 0.69) when comparing AUC results for each cytokine separately (**a**). After merging, combing IL-2 with IL-8 or TNF reached the highest values (AUC = 0.78, each). Combination of three cytokines resulted in an AUC of 0.81. Numbers of samples investigated: SFN patients = 52; controls = 21. *AUC* area under the curve, *IL*− interleukin, *ROC* receiver operating characteristic, *TNF* tumor necrosis factor-alpha

The total AUC of the cytokines IL-2, IL-8, and TNF to distinguish between patients and healthy controls was > 0.5, but remained < 0.7 when investigating single cytokines separately (Fig. 2a, Table 2). A combination of two cytokines resulted in an AUC = 0.74 (for IL-8 and TNF) and an AUC = 0.78 for IL-2 and IL-8 or IL-2 and TNF (Fig. 2b, Table 2). We achieved the highest AUC = 0.81 by combining IL-2, IL-8, and TNF and reached a sensitivity of 70% together with 86% specificity (Fig. 2c, Table 2). When comparing AUC, individual cytokines and the majority of combinations of two or three cytokines did not differ in their discriminative efficacy between SFN and healthy controls (Table 3). The combinations of TNF + IL-2 and TNF + IL-2 + IL-8 showed best discrimination between the two groups (Table 3).

**Table 2:** Diagnostic value of cytokines in SFN

Biomarker	AUC (95% CI)	Sensitivity %	Specificity %	Optimal cut-off
IL-2	0.68	70	62	0.69
IL-8	0.69	76	62	0.65
TNF	0.67	52	86	0.73
IL-2 + IL-8	0.78	64	91	0.76
IL-2 + TNF	0.78	54	91	0.81
IL-8 + TNF	0.74	90	48	0.54
IL-2 + IL-8 + TNF	0.81	70	86	0.67

*AUC* area under the curve, *CI* confidence interval, *IL*− interleukin, *SFN* small fiber neuropathy, *TNF* tumor necrosis factor-alpha

**Table 3:** Comparison of different AUCs

Cytokine combination	p-value	95% CI
IL-8 vs. IL-2	0.93	− 0.21–0.22
IL-8 vs. TNF	0.75	− 0.13–0.18
IL-2 vs. TNF	0.88	− 0.19–0.22
IL-8 vs. IL-8 + IL-2	0.14	− 0.22–0.03
IL-8 vs. IL-8 + TNF	0.39	− 0.10–0.04
IL-2 vs. IL-2 + IL-8	0.07	0.68–0.78
IL-2 vs. IL-2 + TNF	0.08	0.68–0.79
TNF vs. TNF + IL-8	0.27	0.67–0.72
TNF vs. TNF + IL-2	0.04	− 0.24–0.00
TNF + IL-2 + IL-8 vs. IL-2 + IL-8	0.46	− 0.39–0.09
TNF + IL-2 + IL-8 vs. TNF + IL-2	0.40	− 0.03–0.07
TNF + IL-2 + IL-8 vs. TNF + IL-8	0.14	− 0.03–0.20
TNF + IL-2 + IL-8 vs. IL-8	0.08	− 0.01–0.25
TNF + IL-2 + IL-8 vs. IL-2	0.05	0.81–0.68
TNF + IL-2 + IL-8 vs. TNF	0.02	0.02–0.26

*AUC* area under the curve, *CI* confidence interval, *IL*− interleukin, *TNF* tumor necrosis factor-alpha

#### Higher expression of TNF and IL-8 in WBC of female SFN patients compared to female controls

**Fig. 3:**
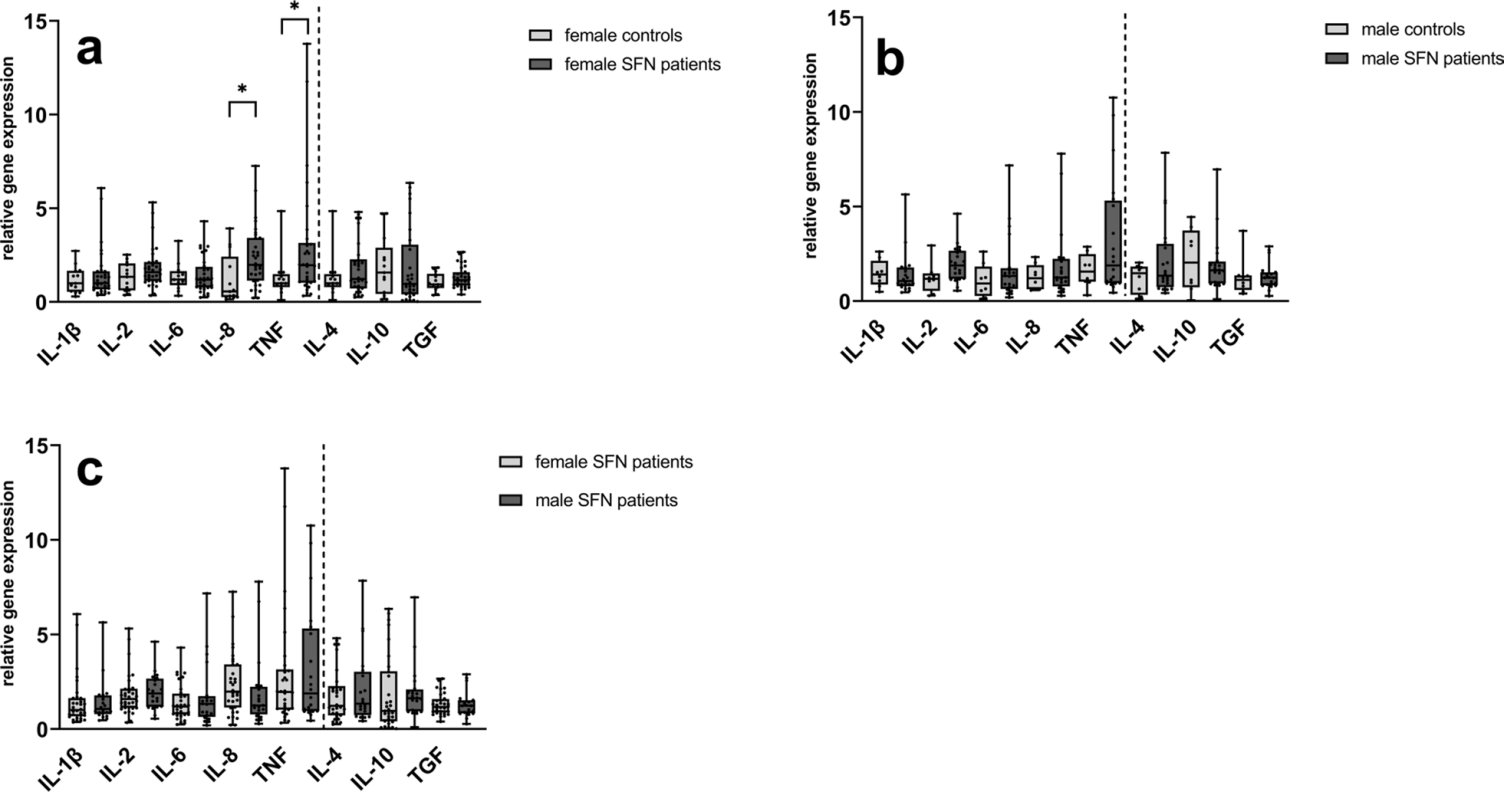
Relative gene expression of cytokines in WBC stratified for sex. The scatter plots show the gene expression of pro- and anti-inflammatory cytokines when comparing **a** female patients and female controls; **b** male patients and male controls; **c** female patients and male patients. Gene expression of IL-8 and TNF was higher in female patients compared to female controls (**a**), no intergroup difference was found comparing male patients **b** and male controls or female patients and male patients (**c**). Numbers of samples investigated: female patients = 32; female controls = 13; male patients = 20; male controls = 8. *IL*− interleukin, *SFN* small fiber neuropathy, *TGF* transforming growth factor beta-1, *TNF* tumor necrosis factor-alpha. *p < 0.05

We found higher gene expression levels of IL-8 and TNF in female SFN patients (n = 32) compared to female controls (n = 13) (IL-8: p = 0.01; TNF: p = 0.03) (Fig. 3a), while we did not detect intergroup differences in male patients (n = 20) and male controls (n = 8) (Fig. 3b). When comparing cytokine levels of female and male SFN patients, we did not find a difference in the gene expression levels of the investigated pro- and anti-inflammatory cytokines (Fig. 3c).

#### Blood cytokine expression profiles differ among SFN patients with moderate and severe pain intensity and among patients with acral and generalized pain

**Fig. 4:**
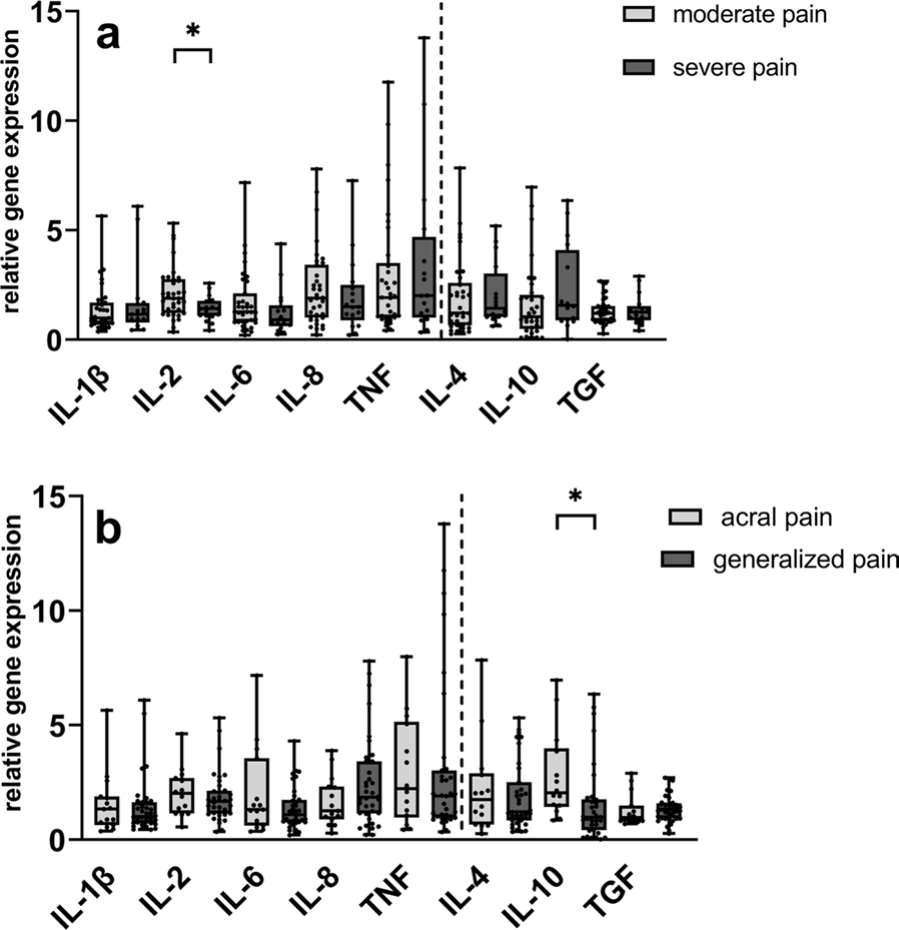
Relative gene expression of cytokines in SFN patients stratified for pain phenotype. The scatter plots show the gene expression of pro- and anti-inflammatory cytokines when comparing patients with moderate (NRS < 4) to severe (NRS ≥ 4) pain **a** and with acral pain to generalized pain (**b**); Gene expression was higher of IL-2 in patients with moderate pain compared to severe pain (**a**) and of IL-10 in patients with acral pain compared to generalized pain (**b**). Numbers of samples investigated: moderate pain = 35; severe pain = 17; acral pain = 15; generalized pain = 21. *IL*− interleukin, *NRS* numeric rating scale, *SFN* small fiber neuropathy, *TGF* transforming growth factor beta-1, *TNF* tumor necrosis factor-alpha. *p < 0.05; **p < 0.01

When stratifying data for pain intensity, we detected higher gene expression levels of IL-2 in patients with no to moderate pain intensity (NRS < 4) (n = 35) compared to patients with severe pain (NRS ≥ 4) (n = 17) (p = 0.02) (Fig. 4a). Dividing the SFN group into patients with acral (n = 15) and generalized pain (n = 21), we found higher gene expression levels of IL-10 in patients with acral pain (p = 0.004) (Fig. 4b) compared to patients with generalized pain. Patients with alternating acral and generalized pain were excluded in this analysis. Thus, the total amount of patients does not reach n = 52.

#### IL-2 gene expression negatively correlates with pain intensity

**Fig. 5:**
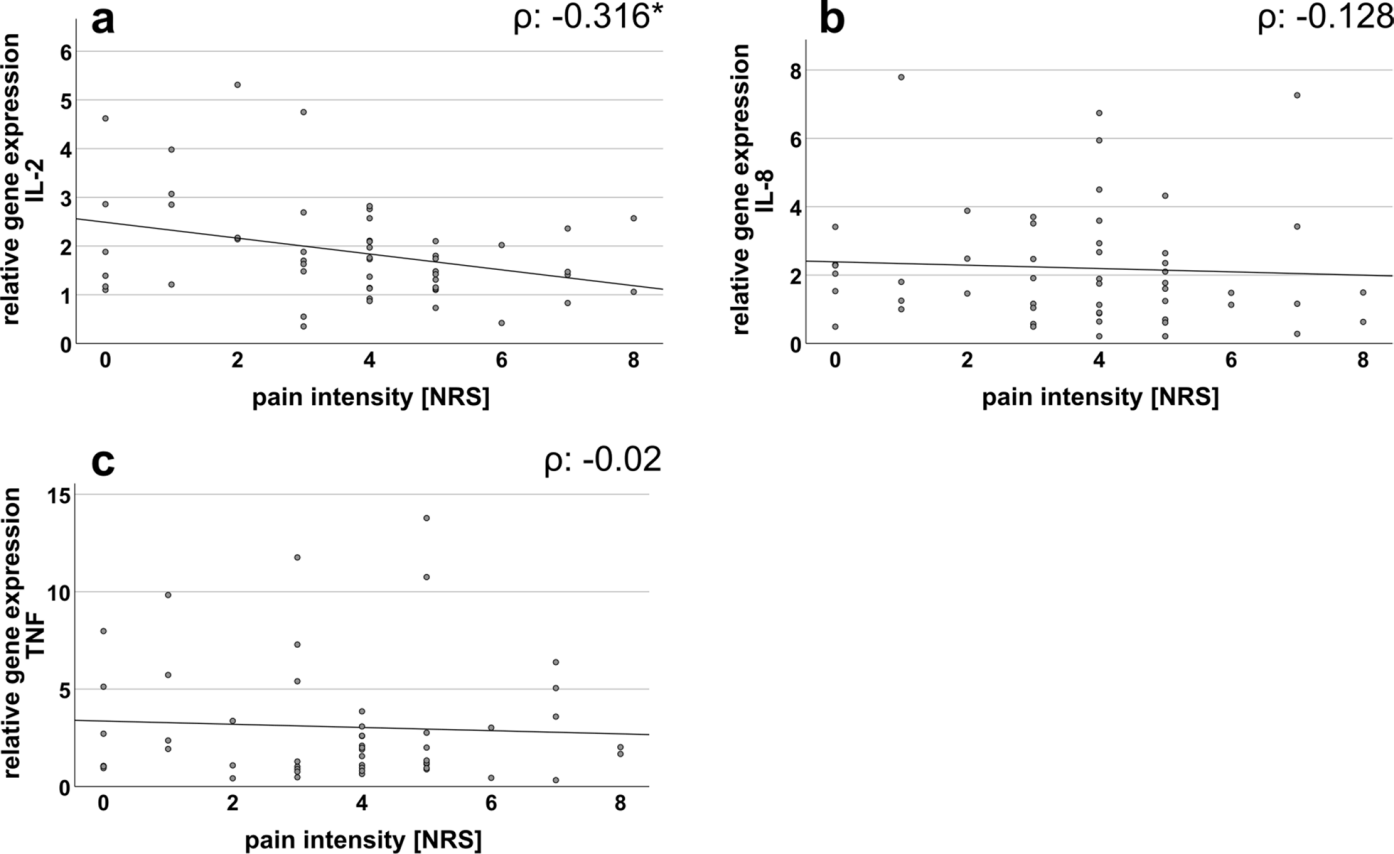
Correlation analysis between WBC cytokine expression profiles and pain intensity in SFN patients. The scatter plots show the gene expression of **a** IL-2, **b** IL-8, and **c** TNF in correlation to the pain intensity (determined on a NRS: range 0 = no pain; 10 = maximum pain imaginable) of SFN patients. Gene expression of IL-2 and current pain intensity correlated negatively in SFN patients (a), while no correlation was found for IL-8 (**b**) and TNF (**c**). Numbers of samples investigated: SFN patients = 52; *IL−* interleukin, *NRS* numeric rating scale, *TNF* tumor necrosis factor-alpha. *p < 0.05

We further found a negative correlation between the relative gene expression of IL-2 and the current pain intensity in SFN patients (p = 0.02) (Fig. 5a). Gene expression of IL-8 and TNF did not correlate with pain intensity (Fig. 5b, c).

### Protein levels of IL-8 and TNF are similar in serum of SFN patients and controls

**Fig. 6:**
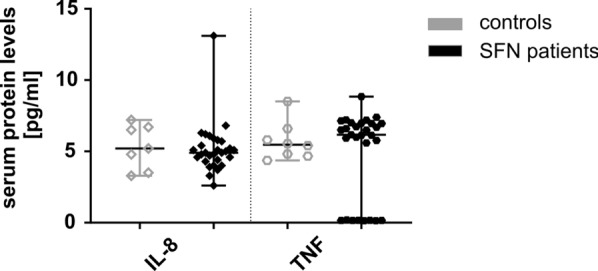
Protein expression of cytokines in serum of SFN patients compared to controls. The scatter plots show the protein expression of pro-inflammatory cytokines IL-8 and TNF in serum of SFN patients and healthy controls. Protein expression of IL-8 and TNF did not differ between groups. Number of samples investigated: SFN patients = 26 (n = 15 female, n = 11 male); controls = 8 (n = 5 female, n = 3 male). *IL*− interleukin, *SFN* small fiber neuropathy, *TNF* tumor necrosis factor-alpha

Protein levels of the pro-inflammatory cytokines IL-8 and TNF did not differ in serum of patients (n = 26; n = 15 female, n = 11 male) compared to controls (n = 8; n = 5 female, n = 3 male) (Fig. 6). IL-2 serum levels remained below the detection thresholds of the ELISA kits used.

## Discussion

We investigated systemic gene and protein expression levels of selected pro- and anti-inflammatory cytokines of SFN patients compared to healthy controls. We found higher gene expression of the pro-inflammatory cytokines IL-2, IL-8, and TNF in patients compared to controls, which also distinguished well between patients and controls when combined.

Cytokines play a major role in the pathogenesis of neuropathic pain [19, 20] and large-fiber neuropathies on a local and systemic level [21–23]. Cytokines are also crucial for activation and recruitment of immune cells and are produced by a wide range of blood cells e.g. monocytes, natural killer cells, peripheral blood mononuclear cells (PBMC), and T-lymphocytes [24]. Their expression patterns vary depending on disease etiology and investigated biomaterial [14, 25, 26].

Previously, we found higher systemic RNA levels of IL-2, TGF, and IL-10 in whole blood samples of SFN patients compared to controls [7]. Here, we show higher IL-2, IL-8, and TNF gene expressions in patients with SFN compared to controls when assessing RNA from the WBC fraction of blood samples, whereas the protein levels of IL-8 and TNF did not differ.

While confirming previous data of an elevated systemic IL-2 gene expression in SFN patients [7], higher systemic IL-2 RNA levels were also found in PBMC of patients with painful polyneuropathies of various etiologies compared to controls [14]. Thus, an elevated systemic IL-2 gene expression is not specific for SFN but may be of potential importance in the development and maintenance of neuropathic pain in SFN.

In a former study, we found higher IL-8 gene expression in fibroblasts obtained from skin punch biopsies of SFN patients [6]. Here, we provide evidence for elevated IL-8 RNA levels also in the WBC fraction. Elevated IL-8 RNA levels in skin and blood cells of SFN patients may indicate a local and systemic inflammatory state. This makes IL-8 an interesting target gene for potential therapeutic means in SFN pending further validation. It is of note that an enhanced expression level of IL-8 was also found in blood, tissue samples, and cerebrospinal fluid of patients with various chronic pain conditions based on small fiber pathology such as burning mouth syndrome [27] or in patients with postherpetic neuralgia [28].

Higher serum TNF expression was previously reported in SFN due to sarcoidosis compared to controls [29, 30]. TNF serum expression was also higher in patients with diabetic polyneuropathy compared to diabetes without polyneuropathy [31]. Further, patients with bortezomib therapy-induced neuropathy showed higher serum levels of TNF in contrast to controls [32]. There are also reports on an analgesic effect of anti-TNF therapy in patients with SFN due to sarcoidosis [29].

Previous studies assessing demographic data and data on pain history of women and men with chronic pain conditions reported equivocal results [33–36]. Some studies found major variations in pain sensitivity and intensity between women and men [33–35]. Others described minor differences with lower thermal and mechanical pain thresholds in women compared to men, while the average pain intensity did not differ between sexes [36]. In line with these data, we found no relevant differences in pain intensity levels between women and men.

One study described a positive correlation between the protein expression of 17 different cytokines (measured in cerebrospinal fluid, plasma, and salvia) and pain intensity in patients with neuropathic pain syndromes [20]. In our previous study, we did not find a correlation between pain questionnaire data (using the short form of the Mc Gill pain questionnaire, the Neuropathic Pain Symptom Inventory, and the Graded Chronic Pain Scale) and cytokine expression in SFN patients [7]. Here, we detected a negative correlation between WBC cytokine gene expression of IL-2 and the current pain intensity, whereas no correlation was found for IL-8 and TNF. With the current study, we provide further evidence for distinct cytokine patterns in patient biomaterial, that may be of use in the clinical management of SFN patients after future mechanistic exploration.

Apart from the pathophysiological role of cytokines, one study provided diagnostic information using serum levels of IL-6, IL-17, and TNF in patients with diabetic neuropathy [13]. In line with our results, the AUC remained below 0.7 for single cytokines and increased after combination of two cytokines to at least 0.7 [13]. Although no single cytokine was likely to show sufficient diagnostic performance in SFN, our findings point towards the potential usefulness of cytokine combinations. To explore clinical utility, further studies including co-influencing parameters and analyzing a larger study cohort are necessary.

In contrast to the results of the gene expression analysis in WBC, we did not find intergroup differences in serum protein levels of the investigated cytokines, which may be due to the small sample size as we confirmed by post-hoc sample size calculation. Another limitation of our study was the restricted subgroup analysis due to the small study cohort. Still, we provide evidence for higher WBC IL-2, IL-8, and TNF expression in SFN compared to controls investigated in a clinically well-characterized study cohort. Further studies are needed to determine the underlying mechanisms potentially linking systemic cytokine expression with SFN pain.

## Conclusions

We found higher systemic gene expression levels of IL-2, IL-8, and TNF in SFN patients, which may be of potential relevance in the development and maintenance of neuropathic pain in SFN. Our data may have implications for accomplishing SFN diagnostics by objective markers and for patient stratification in clinical management and research, which needs further determination in larger patient cohorts.

## Data Availability

All data generated or analyzed during this study are included in this published article. In case of any queries, please contact the corresponding author.

## Abbreviations

A: Absorbance
AUC: Area under the curve
CCM: Corneal confocal microscopy
CI: Confidence interval
CRP: C-reactive protein
EDTA: Ethylenediaminetetraacetic acid
ELISA: Enzyme-linked immunosorbent assay
F: Female
HbA1c: Hemoglobin A1c
IENFD: Intraepidermal nerve fiber density
IL: Interleukin
M: Male
NA: Not applicable
NRS: Numeric rating scale
oGTT: Oral glucose tolerance test
PBMC: Peripheral blood mononuclear cells
PCR: Polymerase chain reaction
PREP: Pain related evoked potentials
QST: Quantitative sensory testing
QSART: Quantitative sensory axon reflex test
Ref.: Reference
RNA: Ribonucleic acid
ROC: Receiver operating characteristic
RT: Room temperature
SFN: Small fiber neuropathy
TGF: Transforming growth factor beta-1
TNF: Tumor necrosis factor-alpha
TSH: Thyroid stimulating hormone
WBC: White blood cells
